# Prediction of molecular subtypes for endometrial cancer based on hierarchical foundation model

**DOI:** 10.1093/bioinformatics/btaf059

**Published:** 2025-02-11

**Authors:** Haoyu Cui, Qinhao Guo, Jun Xu, Xiaohua Wu, Chengfei Cai, Yiping Jiao, Wenlong Ming, Hao Wen, Xiangxue Wang

**Affiliations:** Jiangsu Key Laboratory of Intelligent Medical Image Computing, Nanjing University of Information Science and Technology, Nanjing 210044, China; Department of Gynecologic Oncology, Fudan University Shanghai Cancer Center, Shanghai 200032, China; Jiangsu Key Laboratory of Intelligent Medical Image Computing, Nanjing University of Information Science and Technology, Nanjing 210044, China; Department of Gynecologic Oncology, Fudan University Shanghai Cancer Center, Shanghai 200032, China; Jiangsu Key Laboratory of Intelligent Medical Image Computing, Nanjing University of Information Science and Technology, Nanjing 210044, China; Jiangsu Key Laboratory of Intelligent Medical Image Computing, Nanjing University of Information Science and Technology, Nanjing 210044, China; Jiangsu Key Laboratory of Intelligent Medical Image Computing, Nanjing University of Information Science and Technology, Nanjing 210044, China; Department of Gynecologic Oncology, Fudan University Shanghai Cancer Center, Shanghai 200032, China; Jiangsu Key Laboratory of Intelligent Medical Image Computing, Nanjing University of Information Science and Technology, Nanjing 210044, China

## Abstract

**Motivation:**

Endometrial cancer is a prevalent gynecological malignancy that requires accurate identification of its molecular subtypes for effective diagnosis and treatment. Four molecular subtypes with different clinical outcomes have been identified: POLE mutation, mismatch repair deficient, p53 abnormal, and no specific molecular profile. However, determining these subtypes typically relies on expensive gene sequencing. To overcome this limitation, we propose a novel method that utilizes hematoxylin and eosin-stained whole slide images to predict endometrial cancer molecular subtypes.

**Results:**

Our approach leverages a hierarchical foundation model as a backbone, fine-tuned from the UNI computational pathology foundation model, to extract tissue embedding from different scales. We have achieved promising results through extensive experimentation on the Fudan University Shanghai Cancer Center cohort (*N* = 364). Our model demonstrates a macro-average AUROC of 0.879 (95% CI, 0.853–0.904) in a five-fold cross-validation. Compared to the current state-of-the-art molecular subtypes prediction for endometrial cancer, our method outperforms in terms of predictive accuracy and computational efficiency. Moreover, our method is highly reproducible, allowing for ease of implementation and widespread adoption. This study aims to address the cost and time constraints associated with traditional gene sequencing techniques. By providing a reliable and accessible alternative to gene sequencing, our method has the potential to revolutionize the field of endometrial cancer diagnosis and improve patient outcomes.

**Availability and implementation:**

The codes and data used for generating results in this study are available at https://github.com/HaoyuCui/hi-UNI for GitHub and https://doi.org/10.5281/zenodo.14627478 for Zenodo.

## 1 Introduction

Endometrial cancer is the most common gynecologic neoplasm in developed countries and is the sixth most common cancer in women (Crosbie *et al.* 2022). With the age-standardized incidence rate (ASIR), endometrial cancer has been increasing by an average of 0.69% per year from 1990 to 2019 (Gu *et al.* 2021). Despite a general decline in global endometrial cancer mortality rates, the rising incidence underscores the urgency of strengthening preventive and control measures. In clinical practice, endometrial cancer is usually categorized into Type I and Type II (Bokhman 1983). Type I endometrial carcinomas are mainly low-grade endometrioid tumors that exhibit sensitivity to progesterone and typically have a positive prognosis. In contrast, Type II encompasses a broader range of pathologic subtypes, shows a reduced sensitivity to progesterone, and generally carries a less favorable prognosis. However, this binary categorization is oversimplified and provides limited clinical guidance on risk stratification. With the advent of high-quality molecular data and advancements in pathologic classification, the reliance on traditional pathological diagnosis alone is proving inadequate for effective clinical risk stratification. Therefore, there is an increasing need for more granular and nuanced categorization methods (Suarez *et al.* 2017).

In recent years, a molecularly driven approach to endometrial cancer subtyping has been recommended on top of traditional typing, as proposed by the Cancer Genome Atlas research group (Kandoth *et al*. 2013). This molecular classification later evolved into four molecular subtypes: POLE mutation (POLE mut), mismatch repair deficient (MMRd), p53 abnormal (p53abn), and no specific molecular profile (NSMP). While MMRd and p53abn could be identified by surrogate molecular assay, the POLE mutation would rely on expensive genetic sequencing (Moreira *et al.* 2022). The determination of molecular subtypes is of great clinical significance in guiding patient management. For instance, patients with POLE mut typically have a better prognosis and may benefit from reduced postoperative adjuvant therapy, while those with p53abn have a worse prognosis, necessitating intensive therapy (Stelloo *et al.* 2016). In recent years, molecular subtypes have been increasingly incorporated into clinical practice. Several organizations, including those from the International Federation of Gynecology and Obstetrics (Berek *et al.* 2023), the European Society of Gynaecological Oncology, the European Society for Radiotherapy and Oncology, and the European Society of Pathology (Concin *et al.* 2021), now include molecular subtypes of endometrial cancer in their guidelines.

Despite providing precise and detailed insights into endometrial cancer, the high cost and extended diagnostic time associated with molecular subtyping tests hinder their widespread clinical adoption. Consequently, pathology-based diagnosis remains an essential tool in the diagnostic and treatment decision-making of endometrial cancer, especially in low-resource regions. This indispensable part also inspires us whether we can uncover useful information at the molecular level through the tumor microenvironment. It has been shown that specific morphological patterns presented by tissues are associated with certain genetic alterations among different cancers (Greenson *et al.* 2009, Ninomiya *et al.* 2009, Pai *et al.* 2012). Recent research has showcased the capacity to extract mutation details for diverse cancer categories from whole slide images (WSIs) (Kather *et al.* 2019, Fu *et al.* 2020, He *et al.* 2020, Yamashita *et al.* 2021). This trend is evident in pertinent investigations within the realm of endometrial cancer as well (Hong *et al.* 2021, Darbandsari *et al.* 2024, Wang *et al.* 2024). These findings illustrate the feasibility of identifying disease-related features from microscopic images, offering pathologists new tools to identify imaging markers for diagnosis and prognosis. Existing WSI-based molecular subtyping could be categorized into two groups: classical weakly supervised and multiple-instance learning (MIL)-based pipelines (Ghaffari Laleh *et al.* 2022). Classical weakly supervised analysis pipelines assume all patches of a WSI are equally informative and inherit the slide label, whereas MIL assigns the label only to bags of patches. Fremond *et al.* proposed im4MEC (Fremond *et al.* 2023), an endometrial cancer molecular subtyping pipeline that combines a self-supervised feature extractor with attention-based multiple-instance learning (Attention-MIL). However, the im4MEC requires extremely large batch sizes and hundreds of gigabytes of memory for optimal feature extraction performance. Recently, UNI, a pathology-oriented foundation model (Chen *et al.* 2024) was developed to solve the problem of self-supervised learning. UNI was pretrained on over 1 million diagnostic-grade tissue-stained WSIs of more than 77 TB. It provides possibilities for bypassing the self-supervised training phase and using UNI directly as a pathological feature extractor. However, for domain-specific tasks such as predicting molecular subtypes in endometrial cancer, utilizing a pretrained model within the MIL pipeline may still face challenges in effectively leveraging labeled data to extract the most pertinent information. Fine-tuning a pre-trained network could save the training process and enhance the ability of feature representation for specific tasks (Weiss *et al.* 2016). Previous studies have shown that fine-tuning improves the performance of the pre-trained model with ImageNet weights on specific pathology tasks (Kieffer *et al.* 2017, Faust *et al.* 2019, Riasatian *et al.* 2021). For pathology foundation models like UNI, there is tremendous potential for further performance improvements on specific tasks through fine-tuning.

In this study, we propose a new pipeline for molecular subtyping of endometrial cancer, hierarchical UNI (hi-UNI). The pipeline leverages the Vision Transformer (ViT)-based universal computational pathology model with weakly-supervised and task-specific fine-tuning, inheriting UNI’s prior knowledge and maximizing its domain-specific feature extraction capability. We simplify the molecular subtype prediction task to a weakly supervised classification of tumor patches using soft voting. Additionally, the hierarchical model structure addresses the problem of limited input scale in ViT-based models, better matching the pyramid structure of WSIs. In summary, our model integrates the strengths of traditional weakly supervised methods with the advanced feature extraction ability of UNI, providing an efficient and accurate solution for endometrial cancer molecular subtype prediction.

## 2 Materials and methods

### 2.1 Dataset preparation

**Figure 1.**
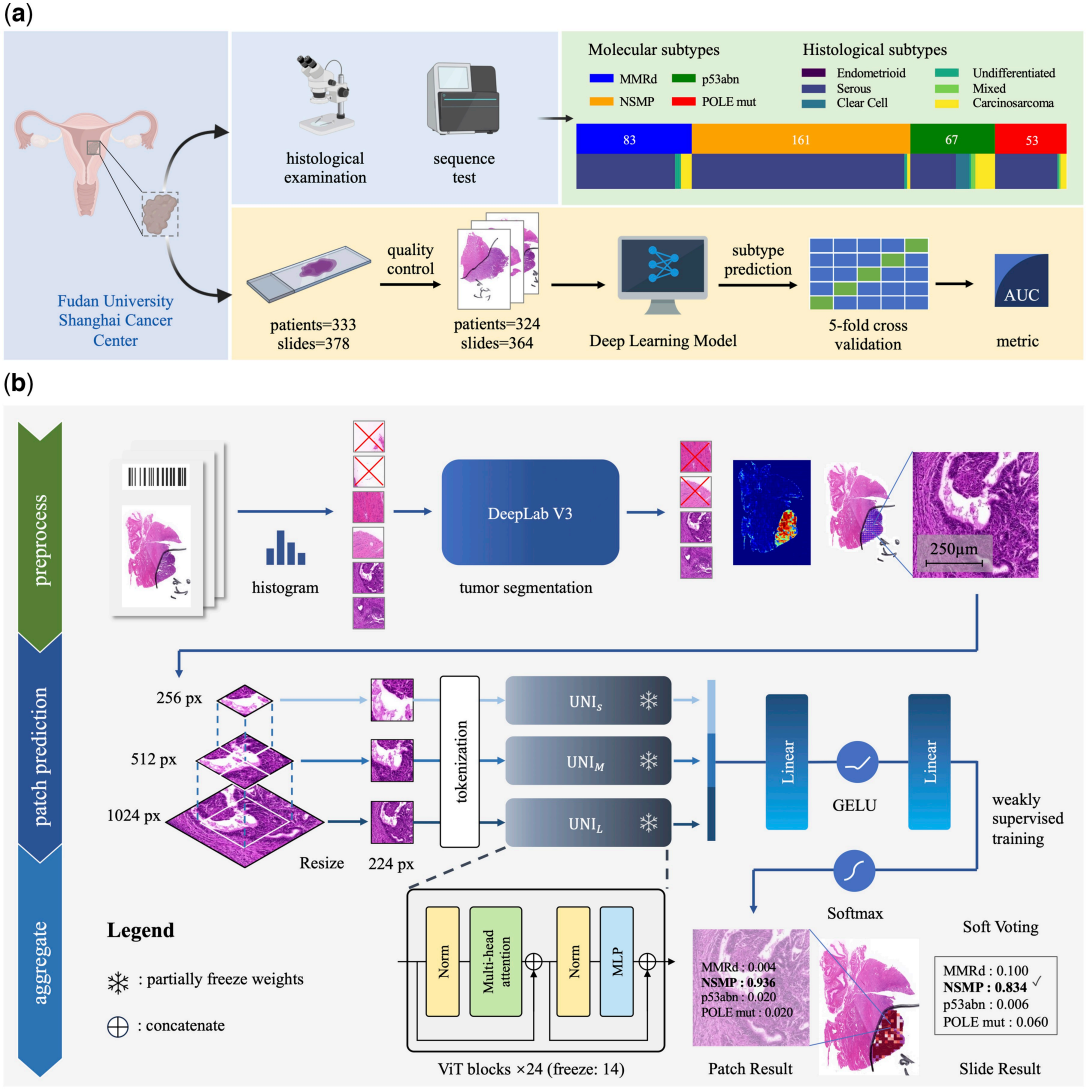
Outline of this study: (a) Overall workflow: data sources, experimental procedures, and validation methods. Created with BioRender.com. (b) Molecular subtype prediction pipeline.

This study collected 378 hematoxylin and eosin (H&E)-stained WSIs from 333 patients with endometrial cancer admitted to Fudan University Shanghai Cancer Center between 2020 and 2023. After screening, samples with incomplete clinical data (*n* = 10, lack of sequencing data necessary for subtype determination) or poor slide quality (*n* = 4, excessive hematoxylin staining or scanning errors) were excluded, resulting in a dataset including 364 WSIs from 324 patients (Fig. 1a). The dataset integrates comprehensive clinical annotations, follow-up patients’ demographics, and high-throughput next-generation sequencing (NGS) data for 46 genes. The cohort contained a total of 83 MMRd slides, 161 NSMP slides, 67 p53abn slides, and 53 POLE mut slides. The molecular subtypes were all obtained by NGS to ensure consistency and accuracy.

### 2.2 Image preprocessing

In the preprocessing stage, the histogram was employed to identify background areas exhibiting excessive luminosity or chromatic intensity. This was achieved by excluding patches with RGB channels’ median values above 200 or below 100. Then we implemented a tumor region segmentation model to minimize the influence of benign and non-tissue regions on model performance. The segmentation model utilized DeepLab v3 (Chen *et al.* 2017), a deep convolutional neural network architecture designed for semantic segmentation. We processed the WSI using a sliding window approach, where each window measured 562 × 562 microns tissue area. The image patches were temporarily down-sampled to 256 × 256 pixels and fed into the DeepLab v3 model, which performed pixel-wise classification to distinguish between tumor and non-tumor regions. The segmentation network was trained using data including detailed annotations of tumor regions on 36 WSIs by pathologists. These annotated slides covered four molecular subtypes and six pathological types, with the model being trained and validated at an 80%-20% split. The network attained an average Dice coefficient of 0.5625 and an average Intersection over Union (IoU) of 0.5401 on the validation set. Ultimately, only patches containing more than 50% tumor regions were selected and saved as 1024 × 1024 patches (0.549 microns per pixel).

Since UNI is based on the ViT architecture, it can only accept inputs of 224 × 224 pixels for tokenization. This means that UNI, with its fixed input size, must choose between high and low magnification when processing images. To adapt the input image patches to the ViT-based model UNI, we performed hierarchical resampling (Fig. 1b) of the selected patches to obtain three different scales: *P_l_*, *P_m_*, and *P_s_* (where *P* stands for patches, and *s*, *m*, and *l* correspond to small, medium, and large resolutions, respectively), as follows:

*P_l_*: Direct down-sampling from a 1024 × 1024 native resolution patch to 224 × 224, which translates to 2.509 microns per pixel. This scale captures macroscopic tissue morphological features, revealing spatial clustering of cells or tissues. It helps analyze organizational structure and distribution patterns over relatively large scales.

*P_m_*: This patch is obtained by cropping the center of a 1024 × 1024 raw resolution patch to 512 × 512 pixels, then down-sampled to 224 × 224, which translates to 1.255 microns per pixel. This scale combines macroscopic histological features and cell-level features, providing a balance between local details and long-distance contextual information.

*P_s_*: We used selective sampling to obtain *P_s_*: The 512 × 512 resolution patch obtained from *P_m_* is cropped into 4 blocks of 256 × 256 resolution. Our proposed pipeline retains the first patch of the four that meets a specific color threshold and discards the entire sample if none exists. The retained patch is then resampled to 224 × 224 to obtain a *P_s_* that translates to 0.627 microns per pixel. This sampling strategy ensures the retention of valid patches with rich microscopic information while minimizing the number of discarded samples. All sampled patches will be preserved for the following steps to ensure the consistency of the experimental results. The detailed flowchart for selective sampling can be found in Supplementary Fig. S2-3.

To address the issue of class imbalance across the four subtypes, we employed undersampling before the k-fold split. Furthermore, the patches belonging to the MMRd and NSMP subtypes were randomly resampled at rates of 70% and 50%, respectively. The final dataset comprises a total of 49 767 patches, with 11 393 MMRd patches (83 WSIs) 15 048 NSMP patches (161 WSIs), 13 599 p53abn patches (67 WSIs), and 9727 POLE mut patches (53 WSIs), which were used for downstream validation.

### 2.3 Model architecture and training process

Subsequently, patches sized at 224 × 224 pixels of varying scales are inputted into three trainable backbone networks, UNI_L_, UNI_M_, and UNI_S_. These networks are all derived from the universal computational pathology foundation model, UNI, and fine-tuned using patches of diverse scales, enabling the simultaneous capture of microscopic cellular details and macroscopic histologic features. To leverage the prior knowledge of the pre-trained foundation model while customizing it for domain-specific tasks, we froze the embedding layer and the first 14 ViT blocks. This approach aims to preserve the model’s generic features while allowing for fine-tuning of subsequent layers to enhance adaptation to the molecular subtyping task. By implementing this freezing strategy, the foundation model can be effectively fine-tuned to new data for downstream tasks, retaining UNI’s feature representations and ultimately enhancing the model’s overall performance and accuracy.

Combining features from the three levels into a consolidated feature vector, we fed this unified vector into linear layers with GELU activation. By merging information from different levels, we generated outputs of four elements representing the four molecular subtypes. This approach culminated in the ultimate classification outcome for the original 1024 × 1024 patch. Through a multi-scale feature fusion step, we harmonized macroscopic histological and microscopic cytological features to comprehensively characterize and classify tumor regions within WSI.

The model utilized weakly supervised learning as it could be trained with only partial or noisy labels, rather than the complete and precise labels required in fully supervised learning. This learning approach assumes that all patches of a WSI inherit its labels, even though some patches may not contain relevant information. During training, patches are randomly sampled from the dataset, and the model parameters are updated using coarse-grained and holistic information.

The experiments used cross-entropy as the loss function and AdamW as the optimizer. The initial learning rate was set to 0.0002, the dropout rate to 0.25, and the batch size to 12. A total of 20,000 iterations were performed, and the model was trained and validated on a single NVIDIA RTX 3090.

### 2.4 Prediction pipeline

In the aggregation phase, patch-level probabilities of the four molecular subtypes were obtained using the softmax activation function through the last linear layer. These probabilities for all image patches were then summed and averaged. The slide-level probability of molecular subtype *j* can be obtained by the following equation:

$$P_{j}=\frac{1}{N}\sum_{i=1}^{N}{\mathrm{Softmax}\left(z_{i}\right)}_{j}$$

where *N* represents the total number of extracted hierarchical patches. *z_i_* denotes the logits from the final linear layer for patch *i*. Softmax (*z_i_*)_*j*_ is the softmax probability for subtype *j* calculated from the logits *z_i_*.

This step calculated each subtype’s probability for the WSI by averaging the probabilities over all patches. This approach more accurately reflected the classes’ characteristics of the WSI, especially in cases where the majority of input patches belong to the tumor region (Ghaffari Laleh *et al.* 2022).

### 2.5 Evaluation metrics and comparative methods

A five-fold cross-validation was used for the validation: 80% of the slides were selected as the training set each time, and the remaining 20% were used as the test set. We use the macro-average or class-wise area under the receiver operating characteristic curve (AUROC) to compare the performance between methods on the validation set. The AUROC can be obtained by the following equation:

$$\mathrm{AUROC}=\int_{0}^{1}\mathrm{TPR}\left(\mathrm{FPR}\right)d\left(\mathrm{FPR}\right)$$

Here, the integral is performed for the change in the false positive rate (FPR) from 0 to 1. The TPR (FPR) is the given true positive rate function under FPR. Accuracy, Precision, Recall, F1 scores, Sensitivity, Specificity, and NPV are also calculated.

A total of three comparative experiments were conducted to test our pipeline for predicting molecular subtypes. First, we compared the patch selections from different tissue regions (tumor versus entire tissue) as the input to our hi-UNI model. Second, we investigated six different ViT block freezing ratios (0%, 20%, 40%, 60%, 80%, and 100%) to validate our selected parameter configuration (60%, equivalent to 14 blocks). Finally, we evaluated the impact of the selective sampling strategy by implementing center-cropping as a preprocessing step for P_s_.

To verify the enhancement of the hierarchical structure of hi-UNI proposed in this paper, we designed five ablation experiments with different combinations of the three hi-UNI scales: combinations of two neighboring scales (UNI_S_+UNI_M_, UNI_M_+UNI_L_) and each scale alone (UNI_S_, UNI_M_, UNI_L_) as outputs.

We also evaluated the performance of our proposed method against several state-of-the-art MIL methods. The comparison included recent methods such as TransMIL (Shao *et al.* 2021), DTFD-MIL (Zhang *et al.* 2022), and SETMIL (Zhao *et al.* 2022), as well as classic MIL methods like CLAM-SB (Lu *et al.* 2021) and Attention-MIL (Ilse *et al.* 2018) (implemented in im4MEC). TransMIL leverages the Transformer’s self-attention mechanism and incorporates a Pyramid Position Encoding Generator (PPEG) to encode spatial information and capture inter-instance correlations, DTFD-MIL addresses small-sample challenges through the introduction of pseudo-bags, and SETMIL employs spatial-encoding-transformer layers to update instance representations, accounting for the multi-scale characteristics of WSIs. The above-mentioned MIL-based approaches were implemented with their default parameters, utilizing entire tissue regions without tumor segmentation. To further evaluate the impact of the tumor segmentation network on MIL methods, we conducted additional experiments where only the tumor regions were used as inputs to these MIL-based approaches. All comparison experiments use the ImageNet (Deng *et al.* 2009) pre-trained ResNet50 model and UNI as feature extraction networks. To evaluate the feature extraction ability of the backbone network, we use t-SNE (t-distributed stochastic neighbor embedding) to visualize the feature vectors extracted from the validation set. The t-SNE can intuitively reflect the backbone network’s ability to distinguish between different subtypes of feature vectors by mapping feature vectors of high dimensional to lower dimensions for visualization. Four visualizations are performed: initial UNI and UNI after weakly-supervised learning fine-tuning, and initial hi-UNI and hi-UNI after weakly-supervised learning fine-tuning.

## 3 Results

### 3.1 Prediction of molecular subtypes

**Figure 2.**
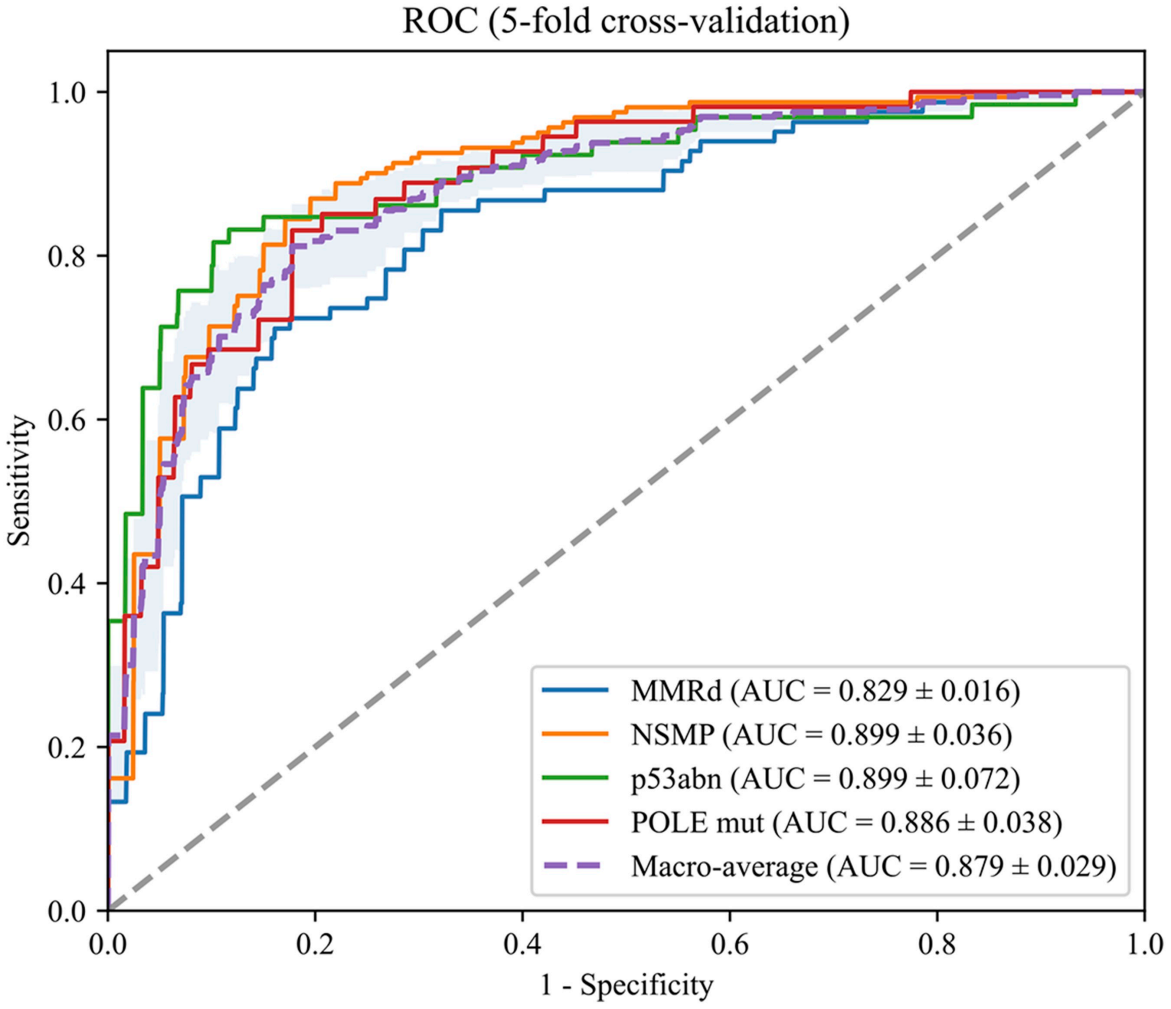
The macro-average and class-wise ROC of the proposed method. Each curve represents the result of interpolated TPR and FPR for each subtype in the five-fold cross-validation.

The ROC of predicting molecular subtypes by the proposed method is shown in Fig. 2. For slide-level prediction, our method achieved a mean AUROC score of 0.829 (95% CI, 0.816–0.843) for MMRd, 0.899 (95% CI, 0.867–0.931) for NSMP, and 0.899 (95% CI, 0.836–0.962) for p53abn, 0.886 (95% CI, 0.853–0.919) for POLE mut. Additionally, a macro-average AUROC of 0.879 (95% CI, 0.853–0.904) was achieved across all subtypes. Detailed accuracy, precision, recall, F1 scores, sensitivity, specificity, and NPV values are reported in the Supplementary Table S1.

The results of the three comparative experiments are provided in the Supplementary data (S2-1, S2-2, and S2-3). Notably, our proposed method, which integrates tumor segmentation, freezes 60% (14) of the ViT blocks and applies selective sampling, achieves the highest performance across all configurations, underscoring the efficacy of its design choices. This could be attributed to the patches from tumor regions, which help the downstream hi-UNI model focus more effectively on subtype-relevant tumor areas. Partial fine-tuning of the original ViT blocks keeps a balance between general pathological knowledge and domain-specific information, while selective sampling further enhances the pipeline by retaining more fine-grained details.

### 3.2 The enhancement of the hierarchical structure

**Figure 3.**
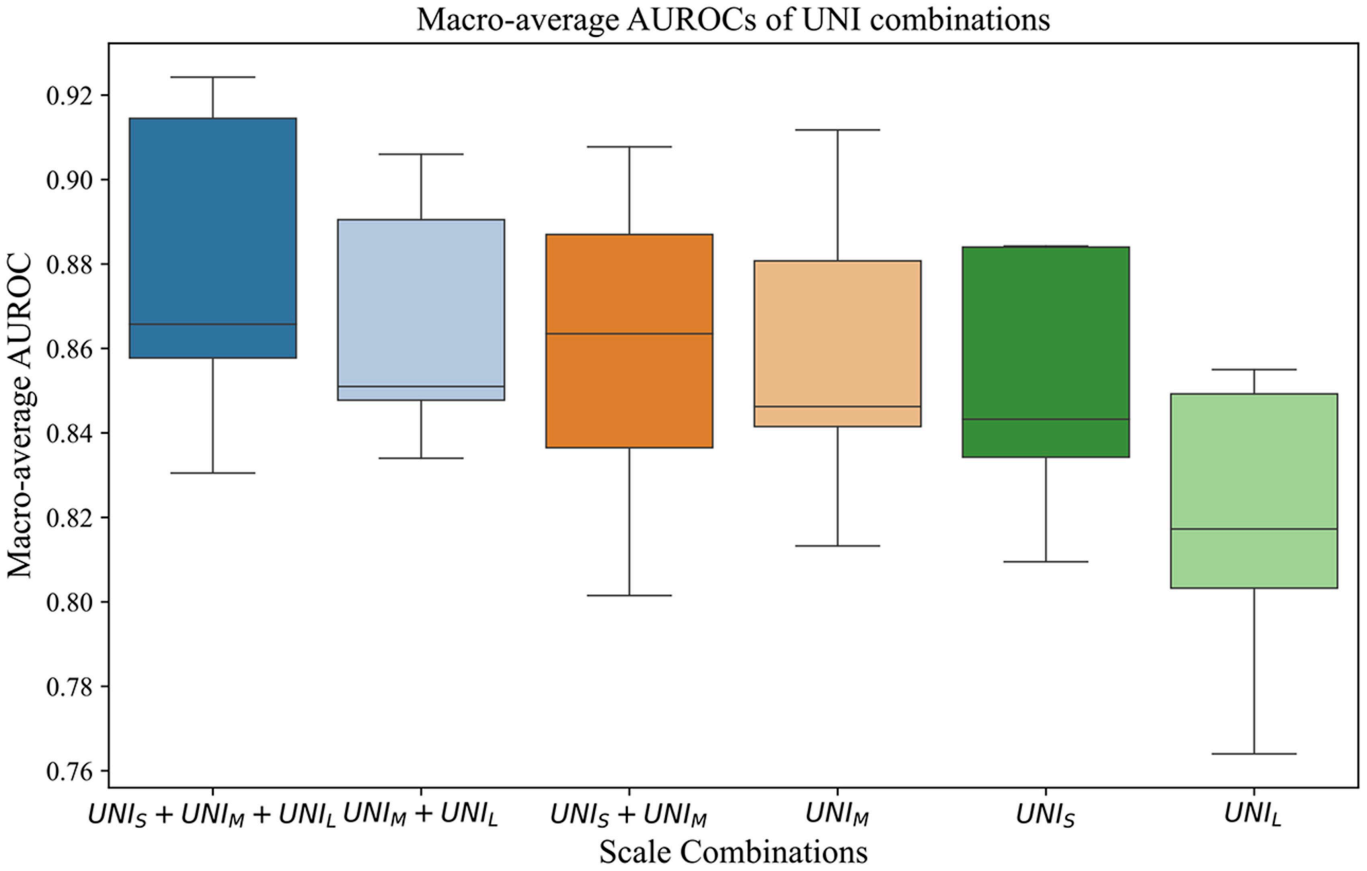
Macro-average AUROC comparison between multiple and single scales from hi-UNI. Boxplots reflect the distribution of AUROCs for five-fold cross-validation.

The results of ablation studies (Fig. 3) demonstrate that the hierarchical structure of hi-UNI positively impacts the molecular subtype prediction task of endometrial cancer, and our proposed hi-UNI with three scales achieved the highest macro-average AUROC of 0.879 (95%CI, 0.853–0.904) in ablation experiments. Two other combinations of neighboring scales (UNI_S_+UNI_M_, UNI_M_+UNI_L_) achieved the suboptimal results of 0.866 (95%CI, 0.836–0.896) and 0.859 (95%CI, 0.831–0.888). The single-scale UNI_M_ yielded a relatively worse result of a macro-average AUROC of 0.858 (95%CI, 0.819–0.896), but higher than the other two single-scale networks, UNI_S_ (macro-average AUROC = 0.851; 95%CI, 0.822–0.880) and UNI_L_ (macro-average AUROC = 0.818; 95%CI, 0.779–0.858). Detailed experimental results are reported in the Supplementary Table S3.

### 3.3 Comparison of proposed method and MIL with different feature extraction networks

**Figure 4.**
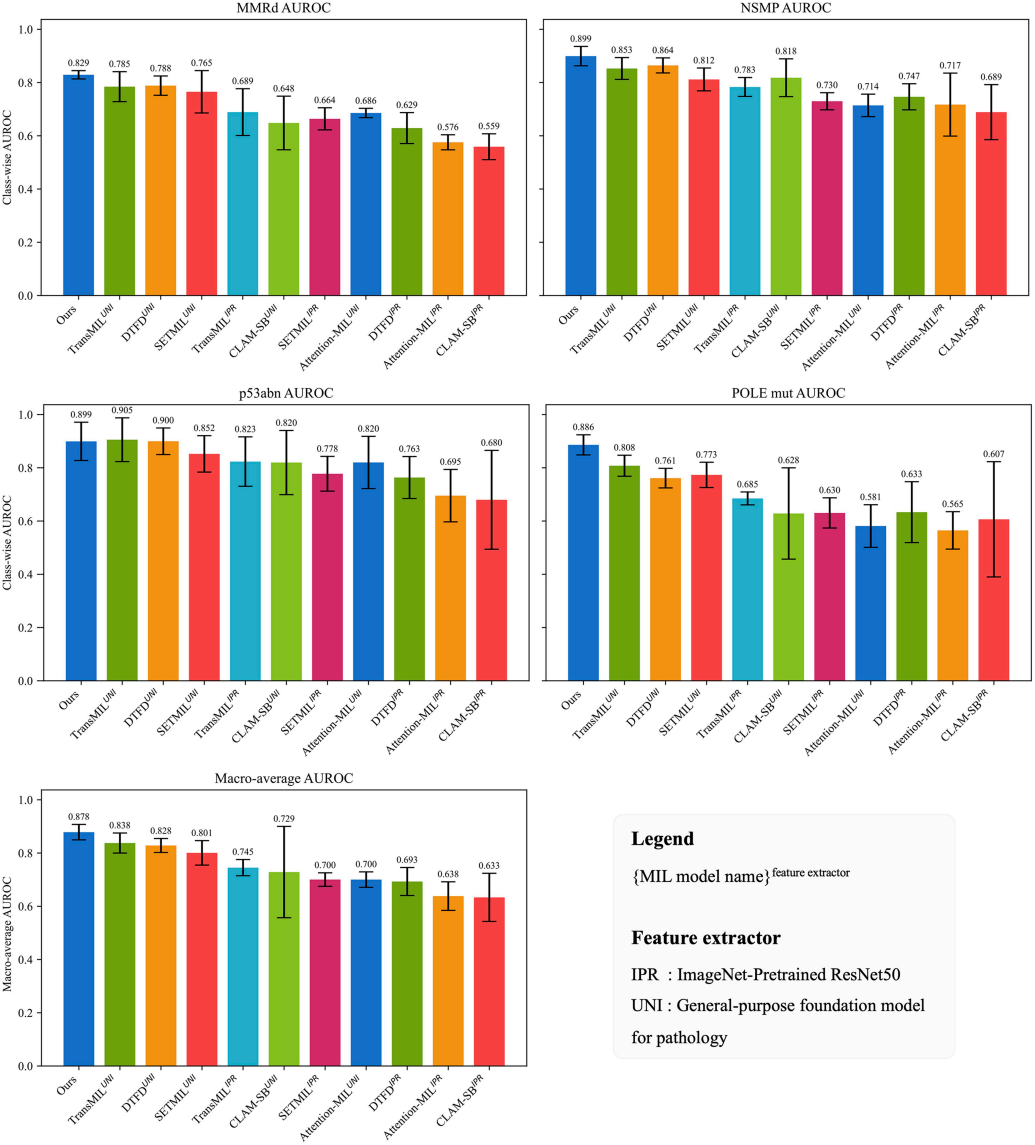
Five-fold cross-validation macro-average and class-wise AUROC comparison of our method and MIL methods with ImageNet pretrained ResNet or UNI as the feature extraction network.

The results (Fig. 4 and Table 1) demonstrate that leveraging UNI as the feature extraction network leads to significant performance enhancements for all the MIL-based methods compared to their counterparts utilizing ImageNet-pretrained ResNet50. In the five-fold cross-validation experiments, TransMIL with UNI demonstrated superior performance among compared MIL methods, achieving an AUROC of 0.838 (95% CI, 0.805–0.871). Our proposed method surpassed TransMIL’s performance on MMRd, NSMP, and POLE mut subtypes, attaining a higher macro-average AUROC of 0.879 (95% CI, 0.853–0.904), though performing marginally lower on p53abn classification. Both DTFD-MIL, which employs pseudo-bags, and SETMIL, which incorporates spatial information, significantly outperformed mainstream approaches like CLAM-SB and Attention-MIL based on both feature extractors. These results underscore the significant performance advancements enabled by UNI over ImageNet-pretrained ResNet as a feature extractor for predicting endometrial cancer subtypes. The results of the additional experiments using MIL methods with the tumor segmentation network are provided in Supplementary Table S4. These findings demonstrate that tumor segmentation networks can also enhance the performance of MIL methods.

**Table 1.** Five-fold cross-validation macro-average and class-wise AUROC comparison of our method and MIL methods with ImageNet pretrained ResNet or UNI as the feature extraction network.^a^

Methods	MMRd	NSMP	p53abn	POLE mut	Macro
CLAM-SB^IPR^	0.559 (0.499–0.619)	0.689 (0.560–0.817)	0.680 (0.449–0.910)	0.607 (0.338–0.875)	0.634 (0.521–0.746)
Attention-MIL^IPR^	0.576 (0.540–0.611)	0.717 (0.570–0.864)	0.695 (0.573–0.818)	0.565 (0.478–0.652)	0.638 (0.572–0.705)
SETMIL^IPR^	0.664 (0.628–0.700)	0.730 (0.702–0.758)	0.778 (0.720–0.835)	0.631 (0.581–0.680)	0.700 (0.678–0.723)
DTFD^IPR^	0.629 (0.578–0.680)	0.747 (0.704–0.790)	0.764 (0.694–0.833)	0.633 (0.533–0.734)	0.693 (0.647–0.739)
TransMIL^IPR^	0.689 (0.612–0.766)	0.783 (0.752–0.815)	0.823 (0.742–0.905)	0.685 (0.664–0.706)	0.745 (0.719–0.772)
CLAM-SB^UNI^	0.648 (0.523–0.773)	0.818 (0.730–0.906)	0.820 (0.670–0.969)	0.628 (0.415–0.841)	0.729 (0.693–0.764)
Attention-MIL^UNI^	0.686 (0.664–0.707)	0.714 (0.662–0.767)	0.820 (0.698–0.942)	0.581 (0.482–0.681)	0.700 (0.664–0.736)
SETMIL^UNI^	0.765 (0.696–0.835)	0.812 (0.774–0.849)	0.852 (0.792–0.912)	0.773 (0.732–0.815)	0.801 (0.760–0.841)
DTFD^UNI^	0.788 (0.757–0.820)	0.864 (0.840–0.889)	0.900 (0.856–0.943)	0.761 (0.729–0.794)	0.828 (0.806–0.851)
TransMIL^UNI^	0.785 (0.735–0.834)	0.853 (0.817–0.889)	**0.906 (0.833–0.978)**	0.808 (0.773–0.842)	0.838 (0.805–0.871)
Ours	**0.829 (0.816–0.843)**	**0.899 (0.867–0.931)**	0.899 (0.836–0.962)	**0.886 (0.853–0.919)**	**0.879 (0.853–0.904)**

IPR, ImageNet-Pretrained ResNet50.

a Bold values indicate the highest value in each column.

**Figure 5.**
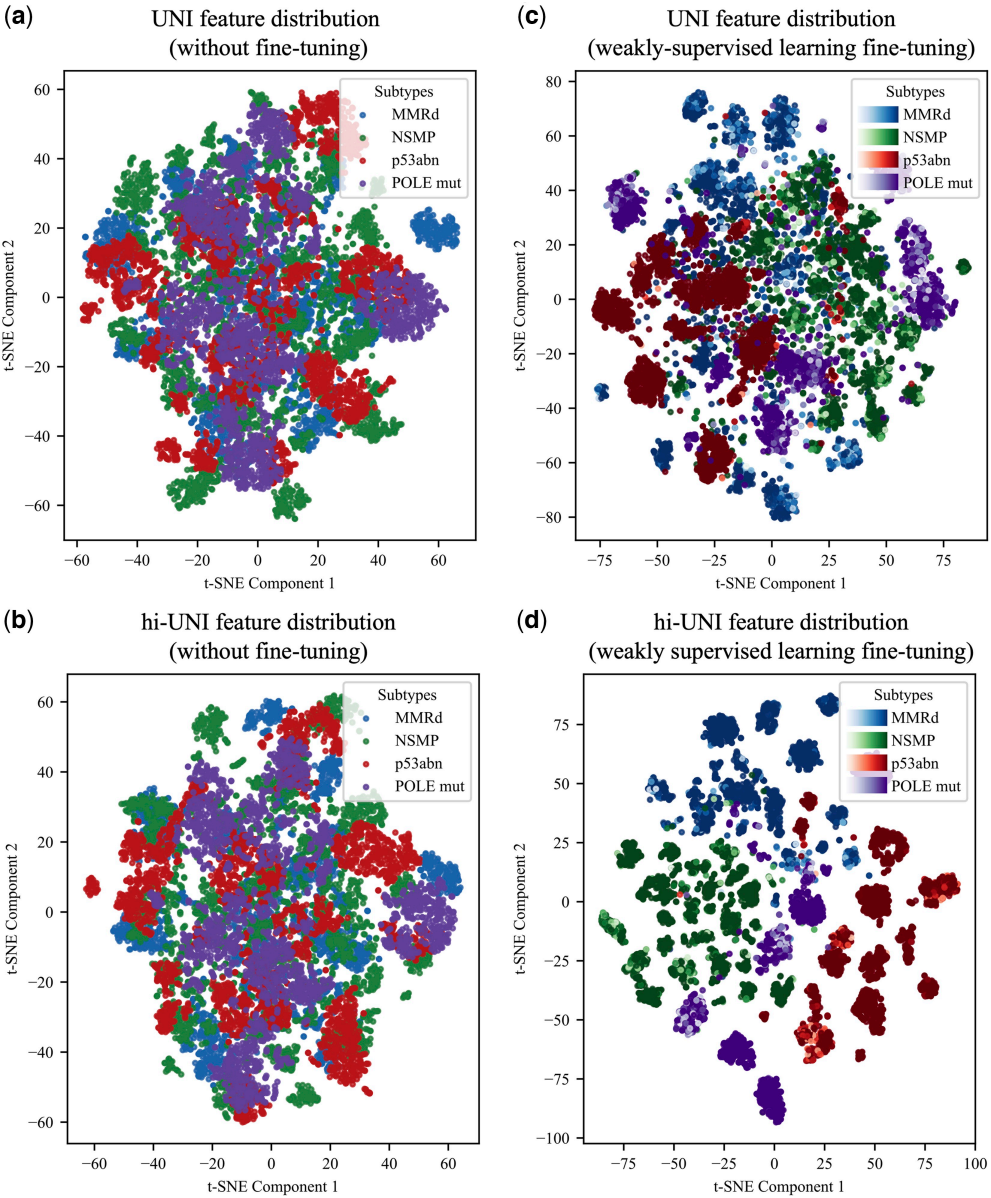
t-SNE visualization of original UNI (without any fine-tuning) and UNI after weakly supervised learning fine-tuning (a, c). t-SNE visualization of original hi-UNI and hi-UNI after weakly supervised learning fine-tuning (b, d). The confidence score of each sample is revealed by its color shades. Single-scale UNI was trained and validated using *P_m_*.

Figure 5a and b shows that the UNI model can distinguish patches’ subtypes including NSMP (green scatters) and p53abn (red scatters) based on its extracted key features but struggles with highly similar patches, as each subtype’s clusters are not yet clearly separated. This is also true for hi-UNI. Figure 5c and d demonstrates that both UNI and hi-UNI models, fine-tuned on the endometrial cancer dataset, significantly enhance feature extraction, where feature vectors corresponding to different subtypes are more distinctly clustered. This indicates that weakly supervised learning could improve the feature representation for classification. The clusters of hi-UNI (Fig. 5d) are more aggregated than those of UNI (Fig. 5c), and the margins of the clusters are more pronounced between the different subtypes, revealing a more prominent subtype-associated profile.

## 4 Discussion

Determining the molecular subtypes of endometrial cancer could significantly enhance clinical patient management. However, the existing sequencing-based methods present challenges in real-world applications due to their extended testing durations and the requirement for advanced facilities. Hence, pathology diagnosis continues to hold paramount importance in the diagnosis and treatment processes for endometrial cancer. To address these challenges, we propose a deep learning method based on a finely tuned hierarchical foundation model capable of directly predicting the four molecular subtypes using standard H&E stained WSI.

For WSI-based classification tasks, MIL-based approaches have been reported to generate excellent results in various cancers, such as diagnosis of colorectal cancer (Yang *et al.* 2022, Wagner *et al.* 2023), breast cancer metastasis detection (Tang *et al.* 2023, Yu *et al.* 2023), and non-small cell lung cancer classification (Lu *et al.* 2021). These methods usually leverage the deep learning model to aggregate image patches into feature vectors and employ the large dataset (i.e. ImageNet) for pretraining the feature extraction network. However, in high-level tasks like molecular subtyping, our method has exhibited superior performance compared to Attention-MIL and CLAM-SB. One of the potential reasons could be the features for distinguishing the pathological subtypes are relatively easier to construct and histomorphological differences between different types have been well summarized (Greenson *et al.* 2009). The ImageNet pretrained feature extraction network could harbor enough image features to drive the diagnostic differentiation. However, identifying morphological distinctions between molecular subtypes is challenging due to the intricate cascade of gene expression processes. For example, in endometrial cancer subtype prediction, no distinct pathological features have been identified that can be applied to molecular subtype classification. Therefore, the ImageNet pre-trained feature extraction model could not effectively capture the key information relevant to molecule-level subtypes. To overcome this challenge, we employ a foundation model pre-trained specifically on large pathological datasets to guarantee that extracted features are histopathology-relevant. As shown in Fig. 4 and Table 1, using the pathology foundation model as a feature extractor significantly enhances MIL methods’ performance of molecular subtyping. Meanwhile, leveraging spatial information or self-attention mechanisms, recent MIL methods such as SETMIL and TransMIL achieve even higher AUROC compared to classic Attention-MIL and CLAM-SB. Integrating tumor segmentation with MIL methods can help narrow this performance gap and improve their overall effectiveness. However, the observed enhancements are less obvious than those achieved through the weakly supervised learning process employed in this study (Supplementary Tables S2-1 and S4). By allowing the weakly supervised learning process to focus specifically on tumor regions, this approach significantly boosts hi-UNI’s performance, achieving a Macro-AUROC improvement of 0.126. Figure 5 further demonstrates feature extraction based on the pathological foundation model could yield consistent improvement regardless of specific model architectures. To further optimize the overall performance of our model, we employ a weakly supervised learning strategy. While self-supervised learning could improve the results for MIL methods, it also requires a huge amount of data and computational resources. In contrast, weakly supervised methods can automatically extract representative information using slide-level labels (Ren *et al.* 2023). To specifically customize our model for molecular subtype prediction, we freeze the embedding layer and the first 14 ViT blocks in the UNI model to preserve the representation ability for generic histopathological features. At the same time, we tune the parameters for subsequent layers to enhance adaptation to the molecular subtyping task. Comparisons between models (Fig. 5) show that weakly supervised learning could continue improving the performance of models for molecule-related prediction.

Hierarchical representations of tissue at multiple resolutions or scales are critical for pathologists to examine the tissue. Incorporating a multiscale structure in deep learning models for pathological tasks lies in the model’s enhanced ability to capture information across different levels of granularity in image features. This allows the model to learn pathological patterns and relationships at different scales effectively. Results from ablation experiments and t-SNE visualization (Figs 3 and 5) consistently show that hi-UNI, using three different scale patches, achieves superior performance compared to the single-scale UNI under the same weakly-supervised training. This verifies that the hierarchical structure of the proposed method can synthesize features from different scale patches to achieve better molecular subtype classification results, addressing the limitation that UNI can only accept pathology images with restricted resolution. Therefore, our model (0.879, 95% CI, 0.853–0.904) outperformed the im4MEC (0.874, 95% CI, 0.856–0.893) by AUROC. Moreover, im4MEC’s results were obtained using self-supervised learning on a dataset containing over two thousand slides and trained on multiple GPUs. In contrast, our approach leverages a simple and effective weakly-supervised learning method that requires only a single GPU to achieve competitive results on relatively smaller datasets, with easier reproducibility.

Our study did have its limitations. Firstly, due to the lack of explicit evaluation metrics for self-supervised learning, we did not strictly follow the self-supervised component of im4MEC. Instead, we used off-the-shelf UNI and ImageNet-pre-trained ResNet50 for reproducible feature extraction. Secondly, we used only one retrospective cohort due to the limited availability of large-scale validation sets with molecular information. A prospective multi-institutional validation could further expedite the implementation of our method in the real clinical setting.

## 5 Conclusion

In this study, we developed a novel hierarchical network integrating weakly supervised learning for endometrial cancer molecular subtype prediction. Our method achieved state-of-the-art performance, offering cost-effective and fast molecular subtyping while presenting a new approach to fine-tuning foundation models for improved feature extraction in computational pathology. This innovation not only advances the utility of foundation models in pathology but also opens avenues for predicting disease subtypes using WSIs.

## Author contributions

Haoyu Cui (Conceptualization [lead], Methodology [lead], Software [lead]), Qinhao Guo (Data Curation [lead], Clinical Expertise [lead], Resources [lead], Validation [lead]), Jun Xu (Conceptualization [equal], Study Design [equal]), Xiaohua Wu (Data Curation [equal], Clinical Expertise [equal], Resources [equal], Validation [equal]), Hao Wen (Data Curation [equal], Clinical Expertise [equal], Resources [equal], Validation [equal]), Chengfei Cai (Investigation [lead], Supervision [supporting], Validation [supporting]), Yiping Jiao (Investigation [equal], Supervision [supporting], Validation [supporting]), Wenlong Ming (Investigation [equal], Supervision [supporting], Validation [supporting]), Xiangxue Wang (Conceptualization [equal], Methodology [equal], Project Administration [lead], Funding acquisition [lead])

## Supplementary Material

btaf059_Supplementary_Data

## Data Availability

The data will be shared on reasonable request to the corresponding author.
