# Supplementary material for: Prediction of molecular subtypes for endometrial cancer based on hierarchical foundation model
Source: Bioinformatics. 2025 Feb 11;41(2):btaf059. doi: 10.1093/bioinformatics/btaf059 (PMC11878776; doi:10.1093/bioinformatics/btaf059)
Supplement: btaf059_Supplementary_Data [file btaf059_supplementary_data.docx]

# Supplementary data

## S1: Comprehensive metrics

AUROC (area under the receiver operating characteristic curve) is used as a unified method for evaluating the performance of different methods. We also report 5-fold cross-validation macro-average accuracy, F1 score, NPV, precision, recall, sensitivity, and specificity in the appendix. The standard deviation (std) is provided.

Table S1 5-fold cross-validation macro-average comprehensive metrics of our proposed method

| Metric | MMRd (macro avg.) | MMRd (std) | NSMP (macro avg.) | NSMP (std) | p53abn (macro avg.) | p53abn (std) | POLE mut (macro avg.) | POLE mut (std) |
| --- | --- | --- | --- | --- | --- | --- | --- | --- |
| Accuracy | 0.818798 | 0.034875 | 0.785845 | 0.036258 | 0.906773 | 0.039067 | 0.879224 | 0.035152 |
| AUROC | 0.829232 | 0.017483 | 0.899309 | 0.040692 | 0.899341 | 0.08039 | 0.886175 | 0.042259 |
| F1 Score | 0.515361 | 0.138322 | 0.792824 | 0.032218 | 0.733862 | 0.11808 | 0.455266 | 0.180157 |
| NPV | 0.852248 | 0.033974 | 0.923045 | 0.045849 | 0.937317 | 0.026613 | 0.899019 | 0.028164 |
| Precision | 0.692647 | 0.148194 | 0.695316 | 0.042639 | 0.756227 | 0.107183 | 0.65619 | 0.230065 |
| Recall | 0.447794 | 0.166224 | 0.925568 | 0.052051 | 0.713187 | 0.128412 | 0.361818 | 0.172536 |
| Sensitivity | 0.447794 | 0.166224 | 0.925568 | 0.052051 | 0.713187 | 0.128412 | 0.361818 | 0.172536 |
| Specificity | 0.929073 | 0.058476 | 0.675366 | 0.069492 | 0.949716 | 0.02018 | 0.967844 | 0.019755 |

These metrics can be calculated using the following formula:

AUROC: the area under the receiver operating characteristic (ROC) curve.

Accuracy = (TP + TN) / (TP + FP + FN + TN)

Precision = TP / (TP + FP);

Recall = TP / (TP + FN)

F1 Score = 2 × P × TPR / (P + TPR);

Sensitivity = TP / (TP + FN);

Specificity = TN / (TN + FP);

NPV = TN / (TN + FN)

where TP means true positive, FP means false positive, FN means false negative, and TN means true negative

## S2-1: Comparative experiment: hi-UNI without tumor segmentation

In this experiment, we excluded only blank patches, retained non-tumor regions, re-extracted the patches, and implemented a 5-fold cross-validation.

Table S2-1 5-fold cross-validation class-wise and macro-average AUROC of the comparative experiment: use hi-UNI without tumor segmentation

| **AUROC\Class** | **MMRd** | **NSMP** | **p53abn** | **POLE mut** | **Macro-average** |
| --- | --- | --- | --- | --- | --- |
| Class-wise-average | 0.662 | 0.764 | 0.838 | 0.745 | 0.753 |
| Std. | 0.058 | 0.049 | 0.0713 | 0.077 | 0.033 |
| 95CI% | (0.612-0.713) | (0.721-0.807) | (0.775-0.900) | (0.678-0.812) | (0.723-0.782) |

## S2-2: Comparative experiment: ViT blocks freeze ratio

In this experiment, we used k-fold cross-validation and performed comparison experiments on different ViT blocks with different freeze ratios (0%, 20%, 40%, 60%, 80%, 100%), which explains the reason for setting the number of blocks frozen to 14 (60%) in this paper.

Table S2-2 5-fold cross validation class-wise AUROC of the comparative experiment: ViT blocks with different freeze ratios (0%, 20%, 40%, 60%, 80%, 100%)

| **Blocks Freeze Ratio (number)** | **Metric** | **Class** | **Mean** | **Std.** | **Avg. AUROC** |
| --- | --- | --- | --- | --- | --- |
| 0% (0) | AUROC | MMRd | 0.813374 | 0.018119 | 0.850399382 |
|  | AUROC | NSMP | 0.864774 | 0.061792 |  |
|  | AUROC | p53abn | 0.900958 | 0.074358 |  |
|  | AUROC | POLE mut | 0.822491 | 0.043115 |  |
| 20% (5) | AUROC | MMRd | 0.832592 | 0.032955 | 0.855495 |
|  | AUROC | NSMP | 0.882567 | 0.058052 |  |
|  | AUROC | p53abn | 0.89055 | 0.086943 |  |
|  | AUROC | POLE mut | 0.816271 | 0.043186 |  |
| 40% (9) | AUROC | MMRd | 0.823925 | 0.028844 | 0.8574655 |
|  | AUROC | NSMP | 0.871345 | 0.044034 |  |
|  | AUROC | p53abn | 0.893852 | 0.064738 |  |
|  | AUROC | POLE mut | 0.84074 | 0.040743 |  |
| 60% (14) | AUROC | MMRd | 0.829232 | 0.017483 | 0.878513962 |
|  | AUROC | NSMP | 0.899309 | 0.040692 |  |
|  | AUROC | p53abn | 0.899341 | 0.08039 |  |
|  | AUROC | POLE mut | 0.886175 | 0.042259 |  |
| 80% (19) | AUROC | MMRd | 0.810626 | 0.026739 | 0.857021 |
|  | AUROC | NSMP | 0.879101 | 0.044565 |  |
|  | AUROC | p53abn | 0.894488 | 0.073709 |  |
|  | AUROC | POLE mut | 0.843869 | 0.040534 |  |
| 100% (24) | AUROC | MMRd | 0.803034 | 0.014491 | 0.846394 |
|  | AUROC | NSMP | 0.862838 | 0.038171 |  |
|  | AUROC | p53abn | 0.885978 | 0.081682 |  |
|  | AUROC | POLE mut | 0.833726 | 0.030414 |  |

## S2-3: Comparative experiment: obtaining P_s_ via center-cropping

In this experiment, we used center-cropping to acquire the topmost patches P_s_ instead of selective sampling, while maintaining consistency with the P_m_ and P_l_ obtained in the original experiment. Subsequently, we implemented a 5-fold cross-validation on the dataset acquired in this way.

Table S2-3 5-fold cross validation class-wise and macro-average AUROC of the comparative experiment: use center-cropping to acquire P_s_ instead of selective sampling

| **AUROC\Class** | **MMRd** | **NSMP** | **p53abn** | **POLE mut** | **Macro-average** |
| --- | --- | --- | --- | --- | --- |
| Class-wise-average | 0.800 | 0.880 | 0.908 | 0.874 | 0.866 |
| Std. | 0.064 | 0.069 | 0.063 | 0.014 | 0.041 |
| 95CI% | (0.744-0.855) | (0.820-0.941) | (0.853-0.963) | (0.862-0.887) | (0.830-0.901) |

Details on selective sampling:

Here, we use three example patches to demonstrate the ability of selective sampling to preserve histogram-threshold-compliant patches (with valid information) at high resolution by sampling up to four times. In contrast, the patches directly obtained from center-cropping are not histogram-threshold-compliant.

Figure S2-3 Detailed flowchart for selective sampling

## S3: hi-UNI’s different scales’ combinations AUROC

Table S3 5-fold cross-validation macro-average AUROC of hi-UNI’s different scale combinations

| **Combinations** | | | **Macro-average AUROC** |
| --- | --- | --- | --- |
| **UNI_S_** | **UNI_M_** | **UNI_L_** |  |
| 🗸 |  |  | 0.851 (0.822-0.880) |
|  | 🗸 |  | 0.858 (0.819-0.896) |
|  |  | 🗸 | 0.818 (0.779-0.858) |
| 🗸 | 🗸 |  | 0.859 (0.831-0.888) |
|  | 🗸 | 🗸 | 0.866 (0.836-0.896) |
| 🗸 | 🗸 | 🗸 | 0.879 (0.853-0.904) |

## S4: Comparison of methods with/without tumor segmentation network

Table S4 Comparison of different methods with/without tumor segmentation network

| **Methods** | **Input: entire tissue region** | **Input: tumor region** | | | | | |
| --- | --- | --- | --- | --- | --- | --- | --- |
|  | Macro | MMRd | NSMP | p53abn | POLE mut | Macro | Macro (change) |
| CLAM-SB^IPR^ | 0.634 (0.521-0.746) | 0.626 (0.471-0.781) | 0.772 (0.668-0.876) | 0.807 (0.734-0.880) | 0.604 (0.578-0.630) | 0.702 (0.617-0.787) | + 0.068 |
| Attention-MIL^IPR^ | 0.638 (0.572-0.705) | 0.769 (0.610-0.695) | 0.755 (0.700-0.810) | 0.807 (0.750-0.864) | 0.712 (0.635-0.789) | 0.732 (0.701-0.762) | + 0.094 |
| SETMIL^IPR^ | 0.700 (0.678-0.723) | 0.667 (0.598-0.737) | 0.756 (0.716-0.795) | 0.795 (0.735-0.854) | 0.645 (0.518-0.773) | 0.716 (0.688-0.743) | + 0.016 |
| DTFD^IPR^ | 0.693 (0.647-0.739) | 0.631 (0.563-0.699) | 0.745 (0.692-0.798) | 0.809 (0.748-0.871) | 0.605 (0.479-0.731) | 0.698 (0.663-0.732) | + 0.005 |
| TransMIL^IPR^ | 0.745 (0.719-0.772) | 0.698 (0.670-0.727) | 0.784 (0.721-0.848) | 0.794 (0.741-0.848) | 0.704 (0.580-0.829) | 0.745 (0.714-0.777) | 0 |
| CLAM-SB^UNI^ | 0.729 (0.693-0.764) | 0.742 (0.629-0.855) | 0.847 (0.805-0.889) | 0.844 (0.773-0.915) | 0.795 (0.630-0.960) | 0.807 (0.760-0.854) | + 0.078 |
| Attention-MIL^UNI^ | 0.700 (0.664-0.736) | 0.769 (0.706-0.832) | 0.866 (0.831-0.901) | 0.889 (0.798-0.981) | 0.737 (0.604-0.869) | 0.813 (0.673-0.953) | + 0.113 |
| SETMIL^UNI^ | 0.801 (0.760-0.841) | 0.796 (0.775-0.818) | 0.844 (0.761-0.926) | 0.896 (0.837-0.956) | 0.829 (0.755-0.903) | 0.841 (0.798-0.884) | + 0.040 |
| DTFD^UNI^ | 0.828 (0.806-0.851) | 0.779 (0.739-0.820) | 0.876 (0.819-0.933) | 0.886 (0.816-0.957) | 0.833 (0.770-0.896) | 0.844 (0.800-0.888) | + 0.016 |
| TransMIL^UNI^ | **0.838 (0.805-0.871)** | 0.820 (0.771-0.868) | 0.874 (0.795-0.952) | 0.880 (0.777-0.984) | 0.762 (0.699-0.826) | 0.834 (0.773-0.895) | - 0.004 |
| hi-UNI | 0.753 (0.723-0.782) | **0.829 (0.816-0.843)** | **0.899 (0.867-0.931)** | **0.899 (0.836-0.962)** | **0.886 (0.853-0.919)** | **0.879 (0.853-0.904)** | **+ 0.126** |
| Note: IPR: ImageNet-Pretrained ResNet50. | | | | | | | |
